# Meat quality prediction using Raman spectroscopy and chemometrics

**DOI:** 10.1186/1758-2946-6-S1-P21

**Published:** 2014-03-11

**Authors:** Marius Nache, Rico Scheier, Heiner Schmidt, Bernd Hitzmann

**Affiliations:** 1 Department of Process Analytics and Cereal Technology, Institute of Food Science and Biotechnology, University of Hohenheim, Stuttgart, 70599, Germany; 2 Research Centre of Food Quality, University Bayreuth, Kulmbach, 95326, Germany

## 

The feasibility of using Raman spectroscopy as a fast and non-invasive method to monitor the quality parameters in pork meat has been investigated. For this application an online prediction methodology has not been established yet. Based on raw Raman spectra of 10 pork *semimembranosus* muscles a range of data pre-processing and multivariate calibration methodology have been used to develop online predictive models for the meat quality parameters: the lactate and pH. The linear and nonlinear algorithms studied were comparatively analysed for speed, robustness and accuracy. Identifying the best “efficiency” evaluation procedure represented the final milestone of the present study. Thus with a cross-validated r^2^ value for both pH and lactate of 0.97, a RMSECV of 4.5 mmol/l for the lactate prediction and 0.06 units for the pH prediction, locally weighted regression provided the most accurate and robust model. This prove the feasibility of using Raman spectroscopy for online meat quality control applications.

